# In Vitro Activity of Isavuconazole and Other Mould-Active Azoles against *Aspergillus fumigatus* with and without CYP51 Alterations

**DOI:** 10.3390/jof9060608

**Published:** 2023-05-25

**Authors:** Michael A. Pfaller, Cecilia G. Carvalhaes, Lalitagauri M. Deshpande, Paul R. Rhomberg, Mariana Castanheira

**Affiliations:** 1 Department of Pathology, University of Iowa College of Medicine, Iowa City, IA 52242, USA; 2 JMI Laboratories, 345 Beaver Kreek Centre, Suite A, North Liberty, IA 52317, USA

**Keywords:** azoles, resistance, surveillance, *Aspergillus fumigatus*

## Abstract

Azole resistance in *Aspergillus fumigatus* (AFM) is mainly associated with mutations in CYP51A and its promoter region or its homologue CYP51B. We evaluated the in vitro activity of isavuconazole, itraconazole, posaconazole, and voriconazole against 660 AFM collected during 2017–2020. Isolates were tested via CLSI broth microdilution. CLSI epidemiological cutoff values were applied. Non-wildtype (NWT) isolates to azoles were screened for alterations in the CYP51 sequences using whole genome sequencing. Azoles had similar activities against 660 AFM isolates. Overall, AFM displayed WT MIC values to isavuconazole (92.7%), itraconazole (92.9%), posaconazole (97.3%), and voriconazole (96.7%). Only 66 isolates (10.0%) were NWT to 1 or more of the azoles, and 32 harbored one or more alterations in the CYP51 sequences. Of these, 29/32 (90.1%) were NWT to itraconazole, 25/32 (78.1%) were NWT to isavuconazole, 17/32 (53.1%) were NWT to voriconazole, and 11/32 (34.4%) were NWT to posaconazole. The most frequent alteration was CYP51A TR34/L98H, carried by 14 isolates. Four isolates carried the alteration I242V in CYP51A, and G448S; A9T, or G138C was carried by one isolate each. Multiple alterations in CYP51A were detected in five isolates. Alterations in CYP51B were noted in seven isolates. Among 34 NWT isolates without -CYP51 alterations, WT rates to isavuconazole, itraconazole, voriconazole, and posaconazole were 32.4%, 47.1%, 85.3%, and 82.4%, respectively. Ten different CYP51 alterations were detected in 32/66 NWT isolates. Alterations in AFM CYP51 sequences can have variable effects on the in vitro activity of the azoles that are best delineated by testing all triazoles.

## 1. Introduction

*Aspergillus fumigatus* is an opportunistic fungal pathogen that is the major cause of invasive aspergillosis (IA) as well as a broad array of chronic and allergic environmentally acquired respiratory diseases [1]. The frequency of IA and associated resistance to the mould-active azole antifungal agents among *A. fumigatus* has increased worldwide over the past two decades [2,3,4,5]. Azole resistance among *A. fumigatus* is mainly associated with mutations in CYP51A and its promotor region or its homologue, CYP51B [3,4,5,6,7]. Resistance to azoles has been shown to develop with prolonged clinical use of azoles in individual patients with chronic bronchopulmonary aspergillosis or through environmental exposure in which azole-naive patients become infected by inhaling conidia that already harbor resistance mechanisms secondary to exposure to azole fungicides [5,8,9]. Despite the detection of azole-resistant *A. fumigatus* throughout the world [5,7,10], most clinical laboratories do not perform antifungal susceptibility testing of *A. fumigatus* or other filamentous fungi [6,11]. As such, there is a paucity of reliable resistance data for isolates of *A. fumigatus* [3,4,5,6,7].

Voriconazole and isavuconazole are the mould-active azoles that are recommended for primary therapy of suspected or documented IA [12,13]. The emergence of azole resistance complicates this process as initial antifungal therapy must be active against *A. fumigatus*. Delays associated with culture, isolate identification, and antifungal susceptibility testing may lead to excess mortality and justify an alternative approach to the use of azole monotherapy [5,12,13,14]. As the frequency of azole-resistant *A. fumigatus* varies widely from country to country depending on local and regional epidemiology, the local rate of resistance, as determined by surveillance, has become the major factor that determines the first line drug therapy for IA [5,6,12,13]. Although routine testing of clinical isolates of *A. fumigatus* is not generally recommended [12,13], periodic assessment of local resistance rates may help guide the management of individual patients [3,4,5,6,7]. It has been suggested that azole monotherapy with either voriconazole or isavuconazole may be used as primary therapy in areas with a low rate of resistance, usually considered to be <5% of isolates, and that MIC testing only be performed in patients failing this therapy [5,12,13]. In regions where higher resistance rates (5–10%) have been documented, routine testing is advisable and azole therapy should be modified as soon as resistance has been detected. If local rates of resistance are greater than 10%, it is recommended that first-line azole monotherapy be discouraged and one of the following treatment regimens be considered: one, voriconazole or isavuconazole in combination with an echinocandin or two, a lipid formulation of amphotericin B or monotherapy with a lipid formulation of amphotericin B [5,12,13,14,15,16,17].

In the present study, we report the MIC distributions for four mould-active azole antifungal agents (isavuconazole, itraconazole, posaconazole and voriconazole) and 660 isolates of *A. fumigatus* sensu stricto that were submitted to the SENTRY Antifungal Surveillance Program (JMI Laboratories, North Liberty, IA, USA) for reference identification and in vitro antifungal susceptibility testing via the CLSI broth microdilution (BMD) method. Isolates submitted for testing were collected in 2017–2020 from clinically significant infections as part of the SENTRY Antifungal Surveillance Program (JMI Laboratories, North Liberty, IA, USA). All isolates were submitted to antifungal susceptibility testing to detect emerging resistance by applying epidemiological cutoff values (ECVs), where available.

## 2. Materials and Methods

### 2.1. Organisms

A collection of 660 non-duplicate clinical isolates of *Aspergillus fumigatus* sensu stricto from the SENTRY Antimicrobial Surveillance Program collected during 2017–2020 were included in the study. Only one fungal isolate per infection episode determined to be significant by local criteria as the reported probable cause of infection were included in this investigation. A total of 40 medical centers in North America (17 sites; 241 isolates), Europe (16 sites; 324 isolates), Latin America (1 site; 11 isolates), and the Asia-Pacific region (6 sites; 84 isolates) have sent isolates to the coordinating laboratory as part of the SENTRY Program.

### 2.2. Identification Methods

Isolates were identified at the participating institutions using methods routinely employed at the submitting laboratory for mould identification [18]. Isolates were submitted to JMI Laboratories (North Liberty, IA, USA) where species identification was confirmed using DNA sequencing and/or proteomic methods [19,20]. Mould isolates were sub-cultured on potato dextrose agar (Remel, Inc., Lenexa, KS, USA) after arrival at the central laboratory and grown for up to seven days to assess purity and viability. Isolates confirmed as pure were inoculated into Sabouraud Liquid Broth, Modified (Becton, Dickenson and Company, Sparks, MD, USA) and the hyphae harvested and prepared for formic acid extraction. Isolates then were submitted to matrix-assisted laser desorption ionization-time of flight mass spectrometry (MALDI-TOF MS) using the MALDI Biotyper (Bruker Daltronics, Billerica, MA, USA). Isolates that did not score ≥2.0 when tested using spectrometry were identified using sequencing of the 28S ribosomal subunit, followed by an analysis of β-tubulin or internal spacer regions (ITS) [19,20,21]). Nucleotide sequences were analyzed using Lasergene^®^ software (DNASTAR, Madison, WI, USA) and compared to sequences using BLAST (https://blast.ncbi.nlm.nih.gov/Blast.cqi; last accessed on 1 May 2021).

### 2.3. Susceptibility Testing

All isolates of *A. fumigatus* were tested via broth microdilution (BMD) using CLSI methodologies [22]. Frozen-form microdilution panels using RPMI 1640 broth supplemented with morpholinepropane sulfonic acid buffer (MOPS) and 0.2% glucose were inoculated with 0.4 − 5.0 × 10^4^ CFU/mL conidial suspensions for a final concentration of 0.2 − 2.5 × 10^4^ CFU/mL. Minimal inhibitory concentrations (MICs) were visualized after 48 h. MIC endpoints were read at the lowest concentration producing visually clear wells. Quality control was performed in accordance with CLSI M38 guidelines using *A. flavus* ATCC 204,304 and *A. fumigatus* ATCC MYA-3626. MIC values were within the QC ranges.

Clinical breakpoints (CBPs) have been published by CLSI for *Aspergillus fumigatus* and voriconazole only (susceptible [S] ≤0.5 mg/L; intermediate [I] 1 mg/L; resistant [R] ≥2 mg/L) (CLSI, 2020a). However, epidemiological cutoff values (ECVs) have been developed for *A. fumigatus* and isavuconazole (ECV, 1 mg/L), itraconazole (ECV, 1 mg/L), posaconazole (ECV, 0.5 mg/L), and voriconazole (ECV, 1 mg/L) [23,24,25]. Isolates for which azole MIC results exceed the ECV were considered non-wildtype (NWT) [23,26]. Whereas the European Committee on Antimicrobial Susceptibility Testing [27] has developed both ECVs (based on MIC distribution) and clinical breakpoints based on MIC distributions, dosing and pharmacokinetic/pharmacodynamic parameters, and likelihood of clinical success and failure, the CLSI has elected at present to provide ECVs but no clinical breakpoints due to a lack of clinical data to support breakpoints [23].

### 2.4. Characterization of Mutations in the Sterol 14 Alpha-Demethylase-Encoding Gene

*A. fumigatus* isolates displaying isavuconazole, itraconazole, posaconazole, or voriconazole MIC values above the ECV (non-wild type [NWT]) were submitted to molecular detection of CYP51A and CYP51B mutations as previously described [19]. Sequences were compared with GenBank sequences available under the accession numbers AAK73659.1 for CYP51A and AAK73660.1 for CYP51B.

## 3. Results

The cumulative frequency of MIC distributions for the four mould-active azoles and *A. fumigatus* are presented in Table 1. Isavuconazole, itraconazole, posaconazole, and voriconazole displayed similar activities (MIC_90_, 1 mg/L, 1 mg/L, 0.5 mg/L, and 0.5 mg/L, respectively; Table 1) against 660 *A. fumigatus* isolates. More than 92.0% of the isolates tested were wildtype (WT) to isavuconazole (92.7% WT), itraconazole (92.9% WT), posaconazole (97.3% WT), and voriconazole (96.7% WT). The overall frequency of NWT strains of *A. fumigatus* was 7.3% for isavuconazole, 7.1% for itraconazole, 2.7% for posaconazole, and 3.3% for voriconazole (Table 1 and Table 2).

The mould-active azoles have been tested against isolates of *A. fumigatus* in the SENTRY Program since 2001 (Table 2). The data from 2001 to 2009 [28] and 2015 to 2017 [29] have been published previously; those data are shown in Table 2 and compared to that of the present study. Whereas the modal MIC values from each time period remained relatively stable for each of the azoles, the percentage of isolates for which the MIC was greater than the ECV (e.g., NWT) increased for each azole over time, indicating a gradual increase in isolates likely to harbor an acquired resistance mechanism. Applying the CLSI clinical breakpoints for voriconazole, the percentage of nonsusceptible isolates (NS; I and R) increased from 4.3% in 2015–2017 to 7.7% in the present study.

Only 66 isolates (10.0% of total) were NWT to one or more of the azoles, 32 of which (48%) harbored one or more alterations in the CYP51 sequences (Table 3). Among the 32 isolates with CYP51 alterations, 25 (78.1%) were NWT to isavuconazole, 29 (90.1%) were NWT to itraconazole, 11 (34.4%) were NWT to posaconazole, and 17 (53.1%) were NWT to voriconazole (Table 3). The isolates withCYP51 alterations were detected most frequently among *A. fumigatus* isolates from Europe (17/324; 5.2%) followed by those from the Asia-Pacific region (4/84; 4.8%) and North America (11/241; 4.6%) (data not shown). None of the isolates from Latin America possessed a substitution in CYP51 sequences. 

The most frequent alteration was CYP51A TR_34_/L98H, carried by 14 isolates from Europe (7 from Italy, 4 from the UK, and 1 each from Belgium, Slovenia, and Germany), all of which were NWT to isavuconazole and itraconazole, 13 were NWT to voriconazole (all 14 were NS via CLSI CBPs), and 8 were NWT to posaconazole (Table 3). Single substitutions in CYP51A were detected in 6/11 isolates from North America, 4 of which carried the alteration I242V (all NWT to itraconazole, all WT to isavuconazole and voriconazole, 3 of 4 WT to posaconazole) (Table 3). One North American isolate carried the CYP51A alteration G448S (NWT to isavuconazole, itraconazole, and voriconazole) and one carried A9T (NWT to isavuconazole). A single isolate from the Asia-Pacific region carried a CYP51A G138C alteration and was NWT to all four azoles.

A series of 3 (F46Y, M172V, E427K) or 5 (F46Y, M172V, N248T, D255E, E427K) alterations on CYP51A were detected in 1 and 3 isolates, respectively; 2 of these isolates were NWT to isavuconazole and itraconazole and 1 was only NWT to itraconazole (Table 3). One isolate from Thailand with CYP51A alterations F46Y, M172V, N248T, D255E, and E427K was only NWT to isavuconazole and harbored the CYP51B alteration Q42L. A single isolate from Belgium was NWT to isavuconazole, itraconazole, and voriconazole and harbored the CYP51A alterations Y121F, M172I, T289A, G448S, and TR46.

Alterations in CYP51B were noted in 7 isolates; 6/7 carried Q42L, 3 from North America (all NWT to itraconazole, and 2 NWT to isavuconazole), 2 from the Asia-Pacific region (NWT to voriconazole or isavuconazole), and 1 from Europe (NWT to isavuconazole, itraconazole, and posaconazole). One of the isolates with the CYP51B alterations K82Q, F149V, and P383L was from Australia and was only NWT to isavuconazole. One of the Asia-Pacific isolates with CYP51B alteration Q42L also contained a series of five mutations in CYP51A (Table 3).

This collection of 660 isolates of *A. fumigatus* contained 594 isolates (90.0%) that were WT to all four azoles, 34 isolates (5.2%) that were NWT to one or more azole but showed no alterations in CYP51A or CYP51B, and 32 isolates (4.8%) that were NWT to one or more azole and harbored alterations in CYP51 (Table 4). Among the 34 NWT isolates without CYP51 alterations, 32.4% were WT to isavuconazole, 47.1% were WT to itraconazole, 82.4% were WT to posaconazole, and 85.3% were WT to voriconazole. By comparison, among the 32 NWT isolates with CYP51 alterations, 21.9% were WT to isavuconazole, 9.4% were WT to itraconazole, 65.6% were WT to posaconazole, and 46.9% were WT to voriconazole.

## 4. Discussion

Antimicrobial resistance (AMR) is an emerging crisis worldwide [3,4,30,31,32]. Whereas most attention is directed towards resistance in bacteria [32,33], antifungal resistance is an undervalued yet important component of AMR [3,4,6,30,31,34]. Recently, both the United States (US) Centers for Disease Control and Prevention (CDC) and the World Health Organization (WHO) have added azole-resistant *A. fumigatus* and *Candida* spp. (*C. auris*) to lists of emerging AMR threats to public health [30,34]. Both organizations have emphasized the importance of conducting standardized surveillance that addresses antifungal resistance and treatability issues.

The SENTRY Antifungal Surveillance Program has been active since 1997 and has included filamentous fungi, including *A. fumigatus*, since 2001 [28,35,36] (Table 2). As such, we have established a baseline database of mould-active azole MIC values for clinical isolates of *A. fumigatus* from hospital locations throughout the world. Data generated in the SENTRY Program may serve as a means of monitoring resistance phenotypes and mechanisms of resistance (MOR) over time and in specific global regions.

The results of the present survey confirm and extend those reported previously from the SENTRY Program [28,29] (Table 2) and other surveillance efforts [3,4,6,10,37,38]. We demonstrated that 90% of *A. fumigatus* isolates were WT to all 4 azoles and, among 66 azole-NWT isolates, 32 harbored one or more alterations in CYP51 sequences (Table 3). The most frequent set of alterations was the so-called environmental mutation CYP51A TR_34_/L98H, detected only in isolates from Europe (Table 3). Although isolates with this set of alterations have been detected in the United States (Berkow et al., 2018), none of the isolates from North America in the present survey possessed these environmental alterations. The TR_34_/L98H alteration resulted in NS (voriconazole; I/R)/NWT MIC results for isavuconazole, itraconazole, and voriconazole, whereas other alterations in *cyp51* can have variable effects on the in vitro activities of the mould-active azoles (Table 3). These effects are best delineated by testing all four azoles. At present, it is unclear that an infection with an *A. fumigatus* isolate that is phenotypically R or NWT (with or without alterations in CYP51) to one azole can be successfully managed using an azole with an S/WT MIC [5,6,8,14,17,19].

An examination of MIC results for azoles and *A. fumigatus* from 2001 through the present showed a gradual increase in the % NWT for isavuconazole (data from 2015 to 2017), itraconazole, and voriconazole (Table 2). Conversely, the % NWT for posaconazole decreased from 3.5% in 2001–2009 to 2.7% in 2017–2020 (Table 2). Application of CLSI CBPs for voriconazole showed an increase in the NS (I/R) percentage from 4.3% in 2015–2017 to 7.7% in 2017–2020 (data not shown). Although the frequency of NWT isolates with alterations in CYP51 was highest in isolates from Europe (5.2%), comparable rates were observed in the Asia-Pacific region (4.8%) and North America (4.6%), suggesting that a decreased susceptibility to azoles is increasing in regions beyond Europe. Indeed, cases with CYP51-mediated resistance have been reported in every continent; moreover, new resistance mechanisms have also been described [3,4,5,6,7]. The prevalence of azole-resistant strains should be investigated in every country in order to understand the prevalence of resistance and adjust therapeutic options where high rates of resistance (>10%) are present [5].

There are some limitations in this SENTRY survey that must be acknowledged. First, we neither collect clinical outcome data nor do we identify those individuals who received an antifungal agent. As such, we are unable to establish any clinical correlation between MIC values and clinical outcomes. Second, we did not identify any mechanisms of resistance beyond alterations in CYP51A/B. There were several isolates of *A. fumigatus* that were NWT to an azole but did not possess specific alterations in CYP51 sequences. The potential for an efflux mechanism accounting for elevated MIC values was not evaluated. Finally, the SENTRY Surveillance Program is a sentinel, not a population-based surveillance.

In summary, the data presented in the present study expand upon the azole MIC distributions for *A. fumigatus*. We noted that the frequency of azole-NWT strains of *A. fumigatus* has increased since a survey conducted in 2001–2009 and now approaches 10% overall, a level at which the use of azole monotherapy is questionable [5]. The azole-NWT isolates harbored alterations in CYP51 that included environmental alterations (e.g., TR_34_/L98H) in isolates from Europe and nonsynonymous point mutations in isolates from North America. Antifungal resistance among isolates of *A. fumigatus* appears to be increasing in North America, Europe, and the Asia-Pacific region. State of the art methods for species identification and antifungal susceptibility testing will be important to further define the impact of azole resistance in both local and regional settings.

## Figures and Tables

**Table 1:** Antimicrobial activity of isavuconazole, itraconazole, posaconazole, and voriconazole tested against *Aspergillus fumigatus*.

Organism (No. of Isolates)	No. and Cumulative % of Isolates Inhibited at MIC (mg/L) of:										MIC_50_	MIC_90_
	≤0.03	0.06	0.12	0.25	0.5	1	2	4	8	>8		
*Aspergillus fumigatus*												
Isavuconazole (660)		0 0.0	3 0.5	49 7.9	422 71.8	138 92.7	26 96.7	13 98.6	5 99.4	4 100.0	0.5	1
Itraconazole (660)			0 0.0	36 5.5	295 50.2	282 92.9	27 97.0	10 98.5	4 99.1	6 100.0	0.5	1
Voriconazole (660)	0 0.0	1 0.2	13 2.1	311 49.2	284 92.3	29 96.7	16 99.1	3 99.5	1 99.7	2 100.0	0.5	0.5
Posaconazole (660)	0 0.0	7 1.1	118 18.9	331 69.1	186 97.3	16 99.7	0 99.7	1 99.8	1 100.0		0.25	0.5

**Table 2:** Frequency of non-wildtype strains of *A. fumigatus* determined via CLSI broth microdilution testing of isavuconazole, itraconazole, posaconazole and voriconazole, 2001–2020.

Year/Antifungal Agent (ref)	No. Tested	Mode (mg/L)	Range	% > ECV
2001–2009 (27)				
Isavuconazole	NA	NA	NA	NA
Itraconazole	1221	0.25	0.015 to >8	2.0
Posaconazole	1312	0.03	0.007 to 2	3.5
Voriconazole	1312	0.25	0.06 to 4	1.4
2015–2017 (28)				
Isavuconazole	1189	0.5	0.12 to 32	3.8
Itraconazole	876	1	0.12 to 32	4.2
Posaconazole	817	0.25	0.008 to 4	2.1
Voriconazole	1122	0.5	0.12 to 32	1.9
2017–2020 (This study)				
Isavuconazole	660	0.5	0.12 to >8	7.3
Itraconazole	660	0.5	0.25 to >8	7.1
Posaconazole	660	0.25	0.06 to 8	2.7
Voriconazole	660	0.25	0.06 to >8	3.3

**Table 3:** Summary of CYP alterations detected among azole non-wildtype *Aspergillus fumigatus* isolates.

Study Year	Site Code	Continent	Country	City	ISC	ITC	VRC	PSC	CYP51A	CYP51B
2018	203	Asia-W. Pacific	Australia	Perth	1	1	2	0.25	wild-type	Q42L
2020	260	Asia-W. Pacific	New Zealand	Auckland	8.1	8.1	8	8	G138C	wild-type
2017	603	Asia-W. Pacific	Thailand	Bangkok	2	1	0.5	0.25	F46Y, M172V, N248T, D255E, E427K	Q42L
2018	131	Europe	Belgium	Antwerp	4	4	2	1	L98H, TR34	wild-type
2019	131	Europe	Belgium	Antwerp	8.1	8	8.1	0.5	Y121F, M172I, T289A, G448S, TR46	wild-type
2018	302	Europe	Czech Republic	Hradec Kralove	2	2	1	0.5	F46Y, M172V, N248T, D255E, E427K	wild-type
2020	91	Europe	France	Caen Cedex	4	4	1	1	wild-type	Q42L
2018	377	Europe	Italy	Milan	8	8	2	1	L98H, TR34	wild-type
2018	377	Europe	Italy	Milan	8.1	8.1	8.1	4	L98H, TR34	wild-type
2018	377	Europe	Italy	Milan	4	4	2	1	L98H, TR34	wild-type
2018	377	Europe	Italy	Milan	4	4	1	0.5	L98H, TR34	wild-type
2018	377	Europe	Italy	Milan	4	4	2	1	L98H, TR34	wild-type
2019	377	Europe	Italy	Milan	4	2	2	0.5	L98H, TR34	wild-type
2019	377	Europe	Italy	Milan	2	2	2	0.5	L98H, TR34	wild-type
2019	329	Europe	Slovenia	Ljubljana	4	8.1	2	0.5	L98H, TR34	wild-type
2019	303	Europe	UK	Leeds	4	8.1	2	0.5	L98H, TR34	wild-type
2020	303	Europe	UK	Leeds	4	4	2	1	L98H, TR34	wild-type
2020	303	Europe	UK	Leeds	4	4	2	0.5	L98H, TR34	wild-type
2020	303	Europe	UK	Leeds	8	8	2	1	L98H, TR34	wild-type
2018	32	North America	Canada	Winnipeg	1	2	0.5	0.5	I242V	wild-type
2018	2	North America	USA	Indianapolis	1	2	1	1	I242V	wild-type
2018	122	North America	USA	Burlington	2	2	1	0.5	F46Y, M172V, E427K	wild-type
2018	806	North America	USA	Richmond	1	2	0.5	0.5	I242V	wild-type
2019	806	North America	USA	Richmond	8.1	8.1	4	0.5	G448S	wild-type
2019	129	North America	USA	New Brunswick	2	2	1	0.5	wild-type	Q42L
2020	122	North America	USA	Burlington	1	2	0.5	0.5	F46Y, M172V, N248T, D255E, E427K	wild-type
2020	456	North America	USA	Birmingham	1	2	0.5	0.25	I242V	wild-type
2020	129	North America	USA	New Brunswick	2	2	1	0.5	wild-type	Q42L
2020	129	North America	USA	New Brunswick	0.5	2	0.5	0.25	wild-type	Q42L
2020	614	Asia-W. Pacific	Australia	Melbourne	2	2	1	0.5	wild-type	K82Q, F149V, P383L
2020	381	Europe	Germany	Hamburg	8	8.1	4	1	L98H, TR34	wild-type
2018	122	North America	USA	Burlington	2	1	0.5	0.25	A9T	wild-type

Abbreviations: ISC, isavuconazole; ITC, itraconazole; VRC, voriconazole; PSC, posaconazole.

**Table 4:** In vitro activity of mould-active azole antifungal agents against azole wild-type (WT) and non-WT (NWT) isolates of *A. fumigatus*.

Azole Phenotype (No. Tested)	MIC_50_	MIC_90_	Range	ECV ^a^	
				%WT	%NWT
WT (594)					
Isavuconazole	0.5	1	0.12 to 1	100.0	0.0
Itraconazole	0.5	1	0.25 to 1	100.0	0.0
Posaconazole	0.25	0.5	0.06 to 1	99.8	0.0
Voriconazole	0.25	0.5	0.06 to 1	100.0	0.0
NWT (no CYP51 alteration) (34)					
Isavuconazole	2	4	0.5 to 8	32.4	67.6
Itraconazole	2	4	0.5 to 8	47.1	52.9
Posaconazole	0.5	1	0.25 to 1	82.4	17.6
Voriconazole	0.5	2	0.5 to 4	85.3	14.7
NWT (with CYP51 alteration) (32)					
Isavuconazole	4	>8	0.5 to >8	21.9	78.1
Itraconazole	2	>8	1 to >8	9.4	90.6
Posaconazole	0.5	1	0.25 to 8	65.6	34.4
Voriconazole	2	4	0.5 to >8	46.9	53.1

Abbreviations: ECV, epidemiological cutoff value. ^a^ CLSI M57S (2022).

## Data Availability

The data presented in this study are available on request from the corresponding author. The data are not publicly available as it is proprietary.
